# Effects of the Presence Rate of the Palmaris Longus Tendon on Wrist Proprioception and Grip Strength

**DOI:** 10.7759/cureus.36779

**Published:** 2023-03-28

**Authors:** Gamze Taskin Senol, İbrahim Kürtül, Gülçin Ahmetoglu, Abdullah Ray

**Affiliations:** 1 Anatomy, Abant Izzet Baysal University Hospital, Bolu, TUR

**Keywords:** proprioception, grip strength, variation, schaeffer’s test, palmaris longus tendon

## Abstract

Introduction: The palmaris longus muscle displays a great variation in terms of incidence and shape. This study has documented the incidence ratio of this muscle among the students at Bolu Abant İzzet Baysal University, Faculty of Medicine, and has revealed the effects of its presence on wrist proprioception and grip strength.

Methods: A total of 101 students between the ages of 18 and 25 were included in the study. Age, height, weight, body mass index (BMI), and dominant upper extremity of the individuals were recorded. After the presence of palmaris longus tendon (PLT) was determined by using the Schaeffer's test, wrist proprioception was evaluated by using a digital inclinometer, and grip strength was evaluated by using a hand dynamometer.

Results: PLT absence rates were evaluated separately as right and left, and it was found as 16.8% and 17.8%. No correlation was found between the dominant upper extremity and BMI and the presence of PLT. The presence or absence of PLT has no effect on grip strength and wrist proprioception.

Conclusion: PLT is used in many clinical areas, such as reconstructive and cosmetic surgery, graft applications, tendon repairs, ptosis correction operations, and ligament stabilization. We think there will be no significant loss in the sense of proprioception and grip strength in the absence of PLT.

## Introduction

The palmaris longus muscle on the region of the forearm is a spindle-shaped tendinous muscle. It starts from the medial epicondyle and attaches to both palmar aponeurosis and flexor retinaculum [1]. It helps wrist flexion functionally and stretches the palmar aponeurosis. Its innervation is provided by the median nerve, and its blood supply is provided by the ulnar artery and its branches [2].

Upon displaying a great deal of variation in the human body and among populations, the muscle may be double, split, tendinous, reverse, or may not be present at all [3]. The incidence of the tendon also differs among populations [4]. It has widely been used in orthopedic, reconstructive, and cosmetic surgeries, such as tendon repairs, ptosis correction operations, ligament stabilization, lip and eyelid reconstruction, and facial paralysis surgery, pointing out its clinical value [3-7].

The literature has grouped senses as exteroceptive, interoceptive, and proprioceptive [7]. Proprioceptive senses perceive the orientation of the body and extremities in space [8]. Hand grip strength (HGS), which can be determined numerically with a hand dynamometer, is a simple, fast, and reliable method used in the evaluation of muscle strength [9]. With the measurement of HGS, the strength of various muscles in hand and forearm can be determined. In this study, it was evaluated whether the presence or absence of the palmaris longus tendon (PLT) has an effect on grip strength.

When the literature is reviewed, it can be seen that the number of studies on proprioception has increased, while the number of studies evaluating wrist proprioception is insufficient. In addition, no studies were found in which the effects of the rate of PLT presence on wrist proprioception were examined. Therefore, this study has been performed to determine the presence rate of PLT in healthy students at Bolu Abant İzzet Baysal University, Faculty of Medicine, and to find out the effects of the rate of PLT presence on wrist proprioception and HGS.

## Materials and methods

A total of 101 (51 females and 50 males) students between the ages of 18 and 25 at Bolu Abant İzzet Baysal University, Faculty of Medicine, with no history of any kind of upper extremity disease, have voluntarily been included in the study. The study was initiated with the 2022/109 decision numbered permission from Clinical Research Ethics Committee.

Schaeffer's test was used to check for the presence of PLT. During the test, the subjects were asked to bring the thumb and little finger together while the wrist was in the supination and flexion position. In this position, after the tendon emerged medial to the radial flexor carpi, PLT was considered to be present after inspection and palpation. 

While measuring wrist proprioception, the subjects were asked to repeat the predetermined target angle with active movement. Flexion proprioception (FP), extension proprioception (EP), ulnar deviation proprioception (UDP), and radial deviation proprioception (RDP) of the wrist were measured by using a digital inclinometer (IP54-DC106, Yiwu Hot Electronic Co., Ltd., China) with 1° sensitivity. After wrist joint movements were reminded to students, they were asked to close their eyes and perform these movements as they learned. Measurements were repeated three times for both hands, and their means were taken. The device was reset after each measurement, and the measurements were initiated when students were ready.

The HGS values were measured in "kilograms" by using Jamar digital hand dynamometer (Asirnow Engineering Co, Los Angeles, USA). Measurements were repeated three times for both hands, and their means were taken. The device was reset after each measurement, and the measurements were initiated when students were ready.

Age, gender, height, weight, and dominant upper extremities were also recorded. The body mass index (BMI) of each student was calculated.

Statistical analysis

Analyses were conducted with Minitab® 21.2 (64-bit, https://www.minitab.com) package program. The normality distribution of the variables was tested with the Anderson-Darling test. Mean (m) and standard deviation (sd) values were calculated for parametric variables, while minimum (min), maximum (max), and median values were calculated for non-parametric variables. For the analysis of changes resulting from PLT presence, two simple t-tests were used for parametric variables, while the Mann-Whitney U test was used for non-parametric variables. For the analysis of correlations between variables, the Pearson correlation test was used for parametric variables, while the Spearman correlation test was used for non-parametric variables. The chi-square test was used for the analysis of categorical variables. Descriptive statistics for variables were used in graphs and tables.

## Results

The median value of the variable of age was found as 20.00 in both female and male students. The BMI variable was found as 21.33 in female students and 23.83 in male students. Descriptive statistics of the variables and significance values as a result of paired comparison tests are shown in Table 1 and Table 2.

**Table 1:** Descriptive statistics of the variables of the right hand. PLT-R, right palmaris longus muscle tendon; FP, flexion proprioception; EP, extension proprioception; UDP, ulnar deviation proprioception; RDP, radial deviation proprioception; ^0^, degree; HGS, hand grip strength; kg, kilogram; a, p-value as a result of Mann-Whitney U test; b, p-value as a result of two simple t-test; k, median (minimum-maximum); l, mean±standard deviation

	PLT-R				
Variables	Present		Absent		
	n		n		p
Age	84	20.00 (19.00-25.00)^k^	17	20.00 (19.00-23.00)^k^	0.630^a^
BMI	84	22.77±3.29^l^	17	22.93±3.20^l^	0.850^b^
FP (^0^)	84	32.76±4.15^l^	17	32.41±3.88^l^	0.746^b^
EP (^0^)	84	32.70 (21.30-43.80)^k^	17	33.22±3.18^l^	0.751^b^
UDP (^0^)	84	11.00 (8.00-20.00)^k^	17	12.00 (10.00±18.00)^k^	0.052^a^
RDP (^0^)	84	15.00 (10.00-24.50)^k^	17	15.00 (9.00-21.00)^k^	0.771^a^
HGS (kg)	84	32.75 (18.40-61.30)^k^	17	25.60 (15.50-53.60)^k^	0.278^a^

**Table 2:** Descriptive statistics of the variables of the left hand. PLT-L, left palmaris longus muscle tendon; FP, flexion proprioception; EP, extension proprioception; UDP, ulnar deviation proprioception; RDP, radial deviation proprioception; ^0^, degree; HGS, hand grip strength; kg, kilogram; a, p-value as a result of Mann-Whitney U test; b, p-value as a result of two simple t-tests; k, median (minimum-maximum); l, mean±standard deviation

	PLT-L				
Variables	Present		Absent		
	n		n		p
Age	83	20.00 (19.00-25.00)^k^	18	20.00 (19.00-23.00)^k^	0.972^a^
BMI	83	22.83±3.36^l^	18	22.64±2.83^l^	0.825^b^
FP (^0^)	83	35.01±4.62^l^	18	34.19±4.58^l^	0.496^b^
EP (^0^)	83	34.62±3.86^l^	18	32.68±3.83^l^	0.056^b^
UDP (^0^​​​​​​​)	83	11.00 (9.00-21.00)^k^	18	10.75 (8.00-18.00)^k^	0.325^a^
RDP (^0^​​​​​​​)	83	14.62±2.42	18	14.44±2.95^l^	0.786^b^
HGS (kg)	83	31.70 (14.40-56.1)^k^	18	28.00 (18.20-49.20)^k^	0.529^a^

The results of the analyses are shown in Table 3.

**Table 3:** p-values showing the result of the chi-square test between the dominant hand and BMI and PLT presence. PLT-R, right palmaris longus muscle tendon; PLT-L, left palmaris longus muscle tendon; BMI, body mass index; PLT, palmaris longus tendon

		PLT-R	PLT-L
Dominant hand		0.524	0.501
	BMI	0.456	0.356

The dominant upper extremity of the individuals was found as the right hand with a rate of 94.1%; the left hand had a rate of 5.0%, and both had a rate of 1.0%. The rate of PLT total presence was 74.25%. The rate of PLT total absence was 25.74%. When PLT absence rates were evaluated separately as right and left, it was found as 16.8% in the right hand and 17.8% in the left hand.

## Discussion

The palmaris longus is among the muscles showing great variation in the body. Its long and thin tendon passes anterior to the flexor retinaculum [10,11]. The absence of palmaris longus was first mentioned in the work "Colombo, De Re Anatomica Libri" [12].

Yet several textbooks have documented the absence ratio of this muscle as 10%-15% [10,13]. As far as the literature is reviewed, no study has been found in which the effect of PLT presence rate on the sense of proprioception and grip strength of the hand has been examined. For these reasons, the study was conducted to investigate the effect of the presence or absence of PLT on wrist proprioception and HGS in healthy individuals. In general, as a result of this study, no correlation was found between the dominant upper extremity and BMI and the presence of PLT. The presence or absence of PLT has no effect on grip strength and wrist proprioception. Based on this result, it was concluded that the PLT can be used in many clinical and surgical fields.

In a study on the absence of PLT and HGS in literature, 365 healthy students with a mean age of 23 were examined, and the absence of PLT was found in 124 (34%) students. Of these students, tendon absence was found in both hands of 82 (22.5%), while tendon was absent in the right hands of 103 (28.2%) cases and in the left hands of 103 (28.2%) cases. Unilateral tendon absence was found in 42 cases (11.5%) [14]. In the same study, although HGS was higher in the hands with PLT than in the hands with PLT absence, it was found that there was no significant difference in this respect [14].

HGS is very important for performing most of the daily living activities [15]. The effects of factors such as anthropometric factors, including the length of the forearm, circumference measurement of the forearm, gender, hand dominance, height, and BMI, have been reported on hand grip and finger grip strength in literature [16-18]. These studies have mostly been conducted in regions such as the knee, ankle, shoulder, and spine, but very few researches have been performed on the wrist [19-22]. In addition, sensory changes resulting from pathological conditions in these regions have also been accumulating in the literature [8,19].

In a study conducted in South Africa in 2014, 706 individuals were examined, and PLT total absence rate was found as 24.4% [23]. In a study conducted in Iran in 2013, 1000 individuals were examined, and PLT total absence rate was found as 22.8% [24]. In our study, the total absence rate was found as 25.74%. Congenital abnormalities affect between 1% and 2% of live births. Of these, around 10% have upper-limb deformities.

A total of three researches, on the PLT absence rate in the Turkish population, documented the following data: in a study conducted on 490 subjects, the incidence of the unilateral, bilateral, and overall absence of the palmaris longus was 5%, 9%, and 11%, respectively, 8.4% for the right hand and 12.2% for the left hand [25]. In another study performed with 1300 volunteering participants, the PLT absence rate was found as 4.51% for the right hand and 7.04% for the left hand [26]. Yet in the other literature done on 1000 cases, the PLT absence rate was found as 0.4% for the right hand and 0.9% for the left hand [6].

In a multiethnic study, 516 individuals were evaluated, and PLT absence rates were found as 14.9%, 2.9%, and 4.5%, respectively, for Caucasians, Asians, and Afro-Americans [27]. In a study that examined 386 individuals conducted in Egypt, a 50.8% PLT total absence rate was reported as the highest absence rate in literature, and it was found that the right hand had a higher PLT rate when compared with the left hand [28]. In a study that examined 890 individuals in Zimbabwe, the PLT absence rate was found as 1.5%. This rate was recorded as the lowest absence rate in the literature [29]. A summary of the results of the studies is shown in Table 4.

**Table 4:** Results of TBA, APRH, APLH, and APLBH measurements in the literature. TBA, total bilaterally absent palmaris longus tendon; APRH, absence of the palmaris longus tendon in right hand; APLH, absence of the palmaris longus tendon in left hand; APLBH, absence of the palmaris longus tendon in both hands

Literature	n	TBA	APRH	APLH	APLBH
Ertem et al.	365	34%	28.20%	28.20%	22.50%
Venter et al.	706	24.40%	6.94%	7.95%	11.90%
Lahiji et al.	1000	22.80%			
Kose et al.	1300	26.59%	4,51%	7.04%	15.04%
Hiz et al.	1000	-	0.40%	0.90%	15.10%
Soltani et al.		Caucasians; 14.9%	11.80%	12.00%	7.60%
		Asians; 2.9%			
		Afro-Americans; 4.5%			
Raouf et al.	386	50.80%	11.90%	7.80%	31.10%
Gangata et al.	890	1.50%	-	-	0.60%

In our study, while the PLT absence rate was found as 16.8% in the right hand, it was found as 17.8% in the left hand. When our study is compared with the literature, it can be seen that our results are in parallel with the literature if we exclude the studies with the highest and lowest rate.

Limitations of our study can be listed as the fact that the gender difference was not taken into account, the age groups were limited, and the active sports life, which is likely to affect muscle strength, was not questioned. In addition, it is seen that variation states of PLT are also examined in the literature. Variations were not examined in this study. Further studies can be planned to make gender comparisons and examine variational situations. The development of the limbs begins early during embryogenesis. Upper-limb buds can be identified 26 days after fertilization and reach a length of 20 to 22 mm by day 53 of pregnancy. The vast majority of congenital deformities occur between the fourth and eighth weeks of pregnancy. After this time, the already-formed structures grow. Buds are formed by the invagination of the mesoderm under the ectoderm. The cells of the somatic mesoderm will form the muscles, nerves, and vessels. The cells of the so-called lateral plate mesoderm will form the bones, cartilage, and tendons. If the PLT does not develop adequately during embryogenesis, it can be absent. But more studies are needed on this subject; our study does not provide data on embryogenesis. This is one of the limitations of our study.

As a result of the analyses conducted, no statistically significant difference was found between the dominant hand upper extremities and PLT presence. Similarly, no statistically significant difference was found between the BMI of the individuals and PLT presence.

Although it is shown in general as 15% in the textbooks, PLT prevalence shows quite different values regardless of any factors, including ethnic diversity. Even in studies conducted within the same ethnic structure, quite different prevalence values ​​have been found. Even if the percentile changes are small, the situation that emerges when the world population is taken into consideration suggests that data on this topic is intricate, which can lead to potential false conclusions. Since it is seen that the PLT presence or absence rate shows different rates in each ethnic group and sometimes even among the same ethnic groups, the presence or absence of PLT should also be determined by precise detection methods. For these reasons, it is essential that agenesis should be definitively proven for the final outcome before any surgical procedure is performed with PLT. 

## Conclusions

We think that after the presence of PLT is determined, it can be used in many clinical and surgical fields, such as reconstructive and cosmetic surgery, graft applications, tendon repairs, ptosis correction operations, and ligament stabilization, without causing a significant loss in proprioception sense and HGS.
